# Impact of the Serum Level of Albumin and Self-Assessed Chewing Ability on Mortality, QOL, and ADLs for Community-Dwelling Older Adults at the Age of 85: A 15 Year Follow up Study

**DOI:** 10.3390/nu12113315

**Published:** 2020-10-29

**Authors:** Yoshiaki Nomura, Erika Kakuta, Ayako Okada, Ryoko Otsuka, Mieko Shimada, Yasuko Tomizawa, Chieko Taguchi, Kazumune Arikawa, Hideki Daikoku, Tamotsu Sato, Nobuhiro Hanada

**Affiliations:** 1 Department of Translational Research, Tsurumi University School of Dental Medicine, Yokohama 230-8501, Japan; okada-a@tsurumi-u.ac.jp (A.O.); otsuka-ryoko@tsurumi-u.ac.jp (R.O.); hanada-n@tsurumi-u.ac.jp (N.H.); 2 Department of Oral bacteriology, Tsurumi University School of Dental Medicine, Yokohama 230-8501, Japan; kakuta-erika@tsurumi-u.ac.jp; 3 Chiba Prefectural University of Health Sciences, Chiba 261-0014, Japan; mieko.shimada@cpuhs.ac.jp; 4 Department of Cardiovascular Surgery, Tokyo Women’s Medical University, Tokyo 162-8666, Japan; tomizawa.yasuko@twmu.ac.jp; 5 Department of Preventive and Public Oral Health, Nihon University School of Dentistry at Matsudo, Matsudo 470-2101, Japan; taguchi.chieko@nihon-u.ac.jp (C.T.); arikawa.kazumune@nihon-u.ac.jp (K.A.); 6 Iwate Dental Association, Morioka 020-0045, Japan; dai-koku@nifty.com (H.D.); tamosato-dent@k-2inc.jp (T.S.)

**Keywords:** mortality, QOL, ADL, Serum albumin, self-assessed chewing ability

## Abstract

Quality of life (QOL) and mortality are true endpoints of epidemiological or medical research, especially for community-dwelling older adults. Nutritional status and activities of daily living (ADLs) are associated with QOL and mortality. Good oral health status supports a good nutritional status. The aim of this study was to elucidate the complex structure of these important health-related factors. We surveyed 354 healthy older adults at the age of 85. Nutritional status was evaluated by the serum level of albumin. QOL, ADLs, self-assessed chewing ability, serum albumin level, and mortality during the 15 year follow up period were analyzed. Self-assessed chewing ability was associated with QOL and ADLs. Self-assessed chewing ability for slight-hard foods was associated with mortality in men. However, it was not associated with the serum albumin level. The serum albumin level was associated with mortality in women. These results indicate that maintaining good oral function is not enough. Nutritional instruction in accordance with oral function is indispensable for health promotion in older adults. When planning health promotion strategies for older adults, different strategies are needed for men and women.

## 1. Introduction

Super-aging societies face many challenges, such as the use of the social security system to access optimal medical services and health services. These services are required to improve quality of life (QOL) and extend life expectancy [1,2,3]. QOL and life expectancy are multifactorial. Knowledge of nutrition and practice of a healthy diet are considered to be the most important factors affecting the health and quality of life of older adults [4,5]. Nutritional interventions for community-dwelling older adults are effective for the promotion of health [6,7,8].

Quality of life (QOL) and mortality are true endpoints of epidemiological studies or medical research. Several studies have focused on the effect of nutritional status on QOL for subjects with specific diseases [9,10,11]. To the best of our knowledge, no study has investigated the effect of nutritional status on QOL for the community-dwelling older adults.

The effect of nutritional status on mortality in community-dwelling older adults is well documented [12,13,14]. Serum level of albumin, which reflects the nutritional status, is a well-known predictor of mortality [15,16,17,18,19,20]. It is applicable for community-dwelling older adults [21,22]. In addition, nutritional status is associated with activities of daily living (ADLs). Evidence concerning nutritional status and ALD for subjects with specific conditions is also accumulating [23,24,25].

Oral health is an important factor in maintaining a healthy nutritional status. Oral functions, especially mastication, are associated with nutritional status. Food preferences depend on masticatory efficiency [26,27]. Overconsumption of carbohydrate-rich foods affects mortality. Excess intake of carbohydrate-rich food is associated with the consumption of excess processed food and not enough raw healthy food [28,29,30]. Oral functions, are key elements in maintaining a healthy nutritional status. However, a systematic review concluded that further study including demographically diverse samples is necessary [31]. For the evaluation of masticatory function, specific devices have been improved to aid in clinical diagnosis [32]. For epidemiological studies, simple questionnaires have been used. By using simple questionnaires, evidence that oral health affects mortality has been accumulated. However, the follow up period used in such studies was short and the age range of the population studied was broad. 

Nutritional status and oral health may be associated with mortality, QOL, and ADLs. These variables interact with each other. Revealing the complexity of these interactions may lead to better understanding of health-related problems. 

Ministry of Health and Labor in Japan directed the 8020 Data Bank Survey at four prefectures in 1997. The aim of this survey was to gather evidence that older adults with their own 20 teeth are active and healthy. In 2002, a five-year follow up study was conducted at Iwate prefecture located in the northeast of Japan. In this follow up survey, the Short form 36 (SF36) [33,34,35] and the Tokyo Metropolitan Institute of Gerontology Index (TMIG index) [36] were introduced. These questionnaires are validated questionnaires for the evaluation of QOL and ADL. In addition, in 2017, a follow up survey was conducted to investigate the mortality of the participants. 

In this study, by using 15-year follow up data from older adults at the age of 85, we investigated the effect of nutritional status, as evaluated by serum level albumin and self-assessed chewing ability, on IADL, QOL, and mortality. The aim of this study was to elucidate the complex relationships among these important health-related factors. 

## 2. Materials and Methods 

### 2.1. Setting

A 15-year follow-up study was conducted with subjects aged 85 years old (from 2002 to 2017) residing in the 11 districts served by one health center in Iwate Prefecture.

### 2.2. Study Population and Survey Frame

In 1997, Japanese Ministry of Labor and Health directed and supported a survey of 80-year-old people residing in four areas in Japan. The details of the survey are described in our previous report [37]. In 2002, the 8020 promotion foundation, which is an affiliated organization of the Japan dental association, supported a follow up survey. Iwate Prefecture, located in the northern region of Japan, was one of the areas that participated in this survey. The sampling method was cluster sampling, and the sampling frame was a complete count survey for all subjects aged 80 years in 1997 (born in 1917) who resided in nine districts in Iwate Prefecture served by one public health center. Between 1997 and 2002, two villages were newly served by the public health center. Sixty-six subjects residing in the two areas participated in the survey conducted in 2002. 

Based on residential registration, public health nurses visited homes in two districts in which subjects who participated in the survey in 1996 lived and in which 85-year-old individuals lived. Public health nurses recommended that all subjects participate in the survey. Among the 435 subjects, 349 agreed to participate, and 345 completed the survey. The surveys, including an oral examination, blood sampling, a medical interview, and a physical fitness test, were conducted at a meeting place or gymnasium owned by the local government. No institutionalized older people were included in this study. In 2017, the 8020 promotion foundation supported a follow up survey that investigated the survival rate of the participants. In October 2017, public health nurses surveyed the participants’ survival and dates of death using the census register. A follow-up survey was conducted using the resident register with surviving subjects participating in the survey in 2002. Details of the follow up survey were described in our previous report [38].

### 2.3. Questionnaire

#### 2.3.1. Quality of Life (QOL)

Quality of life was evaluated by the short form 36(SF-36). The Sf-36 consists of 36 items. These items are classified into 8 subscales: physical functioning (PF), role physical (RP), bodily pain (BP), general health (GH), vitality (VT), social functioning (SF), role emotional (RE), and mental health (MH).

The values of these subscales were standardized and calculated by a program provided by iHope International (Kyoto, Japan) [33,34,35].

#### 2.3.2. Activities of Daily Living (ADLs)

Instrumental activity of daily living was assessed by The Tokyo Metropolitan Institute of Gerontology index of competence (TMIG index) [36]. TIMG index consists of three subscales/dimensions: self-maintenance (S.M), intellectual activity (I.A.), and social role (S.R.). These subscales consist of 5, 4, and 4 items, respectively. If subjects answered yes or able, one point was given for each item. A low IADL (≤4 points), IA (≤2 points), or SR (≤2 points) score is regarded as declining function [39,40]. The TMIG index has been widely used in epidemiological surveys [41,42,43,44,45].

The items included in these subscales are

S.M.: Using public transportation, shopping, preparing, meals, paying bills, managing deposits
I.A.: Filling out pension forms, reading the newspaper, reading books, becoming interested in a new story or program about health.
S.R.: Visiting friends, being called on for advice, visiting sick friends, talking to young people.

#### 2.3.3. Self-Assessed Chewing Ability 

Self-assessed chewing ability was investigated using the following question about 15 different foods: Can you chew the following 15 foods? The response was a simple dichotomous choice (yes/no). Several epidemiological studies have applied this questionnaire for the evaluation of chewing ability [37,46].

### 2.4. Statistical Analysis

#### 2.4.1. Item Response Theory (IRT)

To calculate the summary score for chewing ability, a three-parameter logistic model of the item response theory (IRT) was applied. In addition, factor analysis by the major factor method with varimax rotation was carried out. Summary scores were calculated within each factor [42,43,44,45,46,47,48,49]. IRT analysis was performed using R ver3.50 with the LTR and irtoys packages. 

#### 2.4.2. Structural Equation Modeling (SEM)

Before performing structural equation modeling (SEM), factor analysis by the major factor method with varimax rotation was carried out. Based on the results of the factor analysis, latent variables were constructed. The models were modified through a comparison with the correction index to improve the fitness of the data. For the evaluation of the fitness, the root-mean-square error of approximation (RMSEA) was used for the goodness of fit index [50]. Factor analysis was carried out using SPSS Statistics ver24.0 (IBM, Tokyo, Japan) and SEM was carried out busing AMOS ver24.0 (IBM, Tokyo, Japan).

#### 2.4.3. Generalized Linear Model

To assess the subscales and items of QOL and IADL, the generalized linear model was applied. The distribution of response and link functions was selected using Akaike’s Information Criterion (AIC). The generalized linear model analysis was carried out using SPSS Statistics ver24.0 (IBM, Tokyo, Japan)

#### 2.4.4. Survival Analysis

Survival rates were calculated using the Kaplan–Meier analysis. A log rank test was used to compare significant differences in survival curves. A Cox proportional hazards model was applied to calculate the hazard ratios. Survival analysis was carried out using SPSS Statistics ver24.0 (IBM, Tokyo, Japan)

### 2.5. Ethics Approval and CONSENT to Participate

Informed written consent was obtained from all of the participants at the baseline survey visit. This study was approved by the Ethics Committee of Tsurumi University School of Dental Medicine (Approval Number: 1515).

## 3. Results

### 3.1. Characteristics of the Subjects Who Participated in the Study

The study population consisted of 138 men and 205 women, who were all aged 85 in 2002. After 15 years, 12 subjects had survived. Their health status was evaluated by blood tests. The results are shown in Supplementary Table S1.

### 3.2. Structure of QOL, ADL, and Self-Assessed Chewing Ability of the Older Adults

#### 3.2.1. Structure of QOL

**Figure 1:**
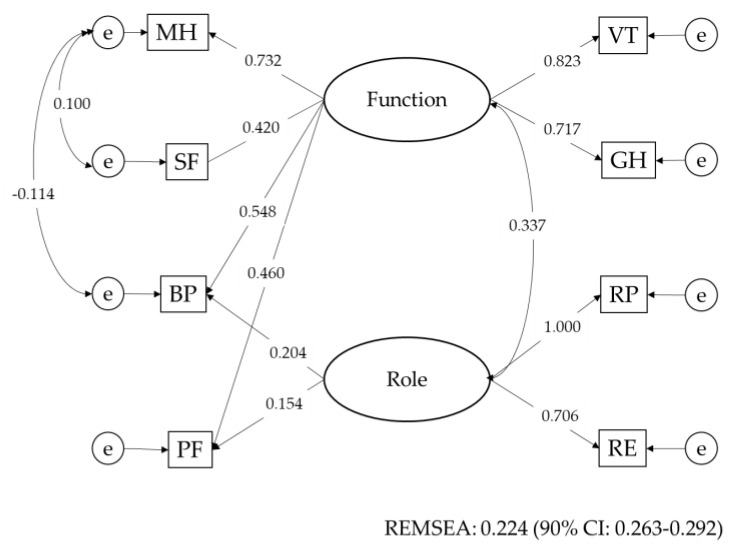
Structure of quality of life of the older subjects at the age of 85. The subscales of the SF 36 consisted of two latent variables, named Function and Role. All paths were statistically significant. BP and PF correlated with both latent variables. Subscales: Physical functioning (PF), Role physical (RP), Body pain (BP), General health (GH), Vitality (VT), Social functioning (SF), Role emotional (RE), Mental health (MH), e: Error variable. SF 36: 36-Item Short-Form Health Survey. REMSEA: root-mean-square error of approximation.

The SF 36 consists of eight subscales. Descriptive statistics of the eight subscales are presented in Supplementary Table S2. For these subscales, factor analysis was carried out through the major factor method with varimax rotation. Factor scores were used as summary scores of the factors for the following analysis. The results are shown in Supplementary Table S3. The subscales consisted of two factors. These factors were named the function and the role. Based on this result, structural equation modeling (SEM) was carried out. The results are shown in Figure 1. Body pain (BP) and physical functioning (PF) correlated with both latent variables.

#### 3.2.2. Structure of ADLs

**Figure 2:**
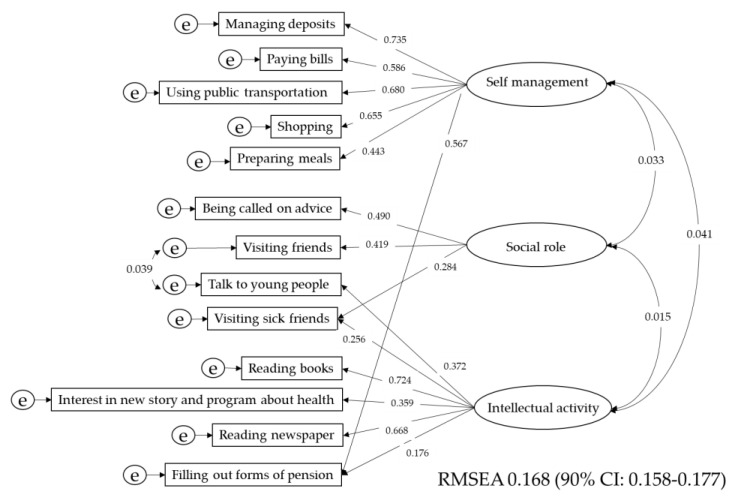
Structure of the ADL. Items of the TIMG Index involved 3 factors. Visiting sick friends and filling out the pension. Pension were correlated with two latent variables. Correlations between latent variables were statistically significant. However, the correlations were very weak. ADL: activity of daily living, TMIG index: The Tokyo Metropolitan Institute of Gerontology index of competence. e: Error variable. REMSEA: root-mean-square error of approximation

The TIMG index consists of three subscales and has a total of 13 items. The scores of these items and the descriptive statistics of the three subscales are shown in Supplementary Tables S4 and S5. For the structure of the IADL, factor analysis and SEM were carried out in the same way as for the QOL. The results of the factor analysis are presented in Supplementary Table S6. Factor scores were used as summary scores of the factors for the following analysis. The model with SEM is shown in Figure 2. Items of the TIMG Index involved three factors. Visiting sick friends and filling out the pension form were correlated with two latent variables. Correlations between latent variables were statistically significant. However, the correlations were very weak.

#### 3.2.3. Structure of Self-Assessed Chewing Ability and Correlation with Number of Remaining Teeth

**Figure 3:**
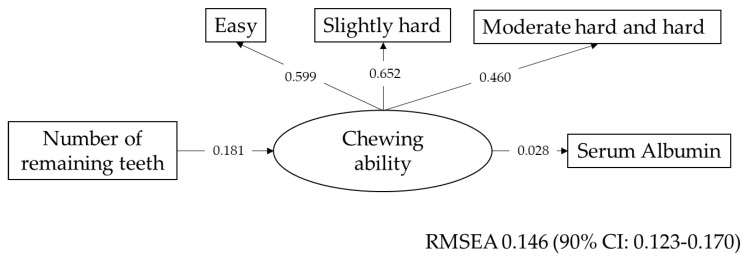
Correlations among number of remaining teeth, chewing ability, and serum level of albumin. REMSEA: root-mean-square error of approximation

Self-assessed chewing ability was evaluated by whether participants were able to chew 15 foods. The variables were dichotomous. To calculate the summary score, the item response theory analysis (IRT) was carried out. The item response curve and item information curves are shown in Figure S1, and the model is shown in Supplementary Table S7. Similar to the QOL and IADL, a factor analysis was carried out for these 15 foods. The results are shown in Supplementary Table S8. The fifteen foods had three factors, and the factors were named easy to chew food, slightly hard to chew food, and moderate and hard to chew foods. The correlations among chewing ability, number of remaining teeth, and serum level of albumin as indicators of nutritional status were analyzed by SEM (Figure 3). The link between chewing ability and serum albumin was not statistically significant (*p* = 0.692). Other than that, all associations were statistically significant.

### 3.3. Interaction of Nutritional Status, and Self-Assessed Chewing Ability with IADL and QOL

#### 3.3.1. Correlation between Self-Assessed Chewing Ability and QOL

A generalized liner model was applied to the dimensions of QOL calculated by the factor scores presented in Section 3.2.1. The results were shown in Table 1. For both function and role, the summary score of self-assessed chewing ability was significantly correlated. However, the number of remaining teeth and serum level of albumin were not statistically significant.

#### 3.3.2. Correlation between Self-Assessed Chewing Ability and IADL

The TIMIG Index consists of three subscales: Self-management, Intercultural activity, and Social role. For these subscales, the generalized liner model was applied. The results were shown in Table 2. The summary score of self-assessed chewing ability calculated by IRT was significantly correlated with the three subscales. However, the number of remaining teeth and the serum level of albumin were not statistically significantly correlated with this factor.

### 3.4. Effects of Nutritional Status, Self-Asssessed Chewing Ability, and IADL on Mortality

To assess the mortality rate at the 15-year follow up, nutritional status evaluated by the serum level of albumin, subscales of self-assessed chewing ability, and the IADL were analyzed using Cox’s proportional hazard model. The results are presented in Table 3. 

For women, only serum albumin level was shown to have a statistically significant effect on mortality, and its hazard ratio was the highest. In contrast, for men, the self-assessed chewing ability of moderate hard food, and intercellular activity had statistically significant effects on mortality. The number of remaining teeth did not have a statistically significant effect. However, when classified as edentulous or dentate, the hazard ratio of edentulous was statistically significant in men (hazard ratio: 1.766, 95% CI; 1.119–2.788, *p* = 0.015). Additionally, hazard ratios of chewing ability were adjusted by health status evaluated by blood tests. Results were shown in Supplementary Table S10. For men, adjusted hazard ratios of self-assessed chewing ability were statistically significant except for Creatinine. 

**Figure 4:**
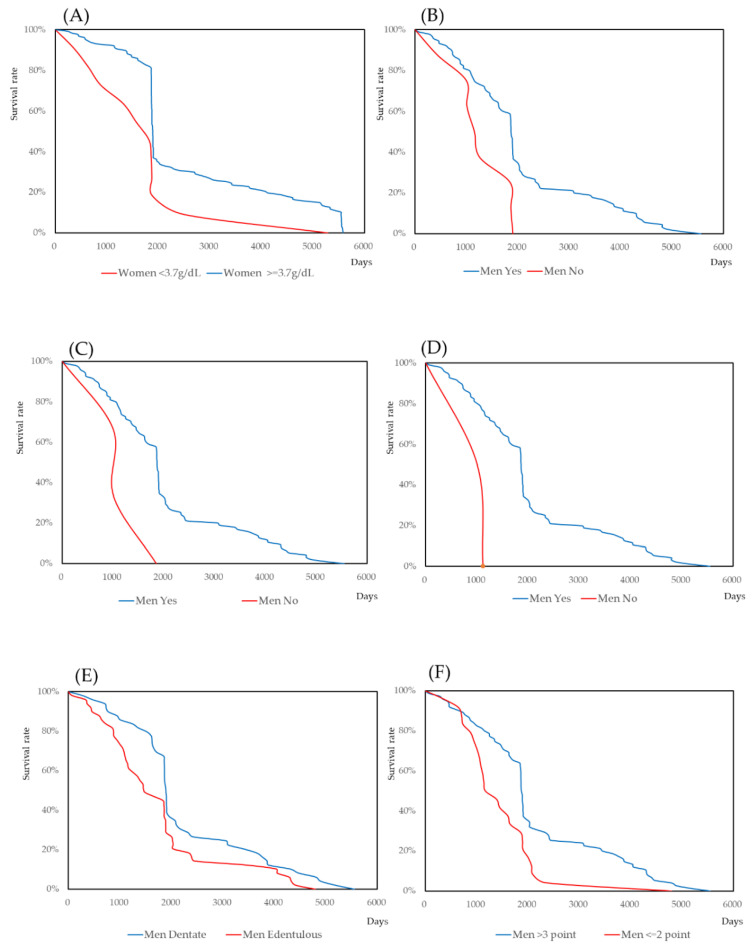
Survival curves of the significant factors for mortality. (**A**) Serum levels of albumin for women. (**B**) Ability to chew Konnyaku-jelly of men. (**C**) Ability to chew Tubular roll of boiled fish paste of men. (**D**) Ability to chew Steamed rice of men. (**E**) Edentulous. (**F**)Intellectual activity of men.

The Kaplan–Meier analysis was used to calculate the survival rate. As self-assessed chewing ability is a contentious variable, the ability to chew three foods (konnyaku jelly, tubular roll of boiled fish paste, and steamed rice) was used as a dichotomous variable. The means and medians of the survival rate are shown in Supplementary Table S11. The survival curves of the statistically significant factors are shown in Figure 4.

### 3.5. Overview of the Interactions Among Health-Related Factors

**Figure 5:**
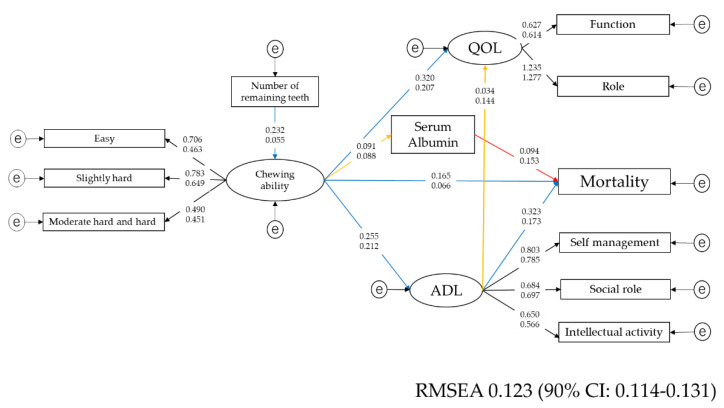
Overview of the interactions among health-related factors. Black lines indicated statistical significance for both men and women, blue lines indicate significance only in men, red lines indicate significance only in women, and orange lines indicated no significance for either men or women. e: Error variable. QOL: quality of live. ADL: activity of daily living. REMSEA: root-mean-square error of approximation.

Finally, by using all health-related factors investigated in this study, multiple group structural equation modeling was conducted for men and women. The results are presented in Figure 5. Black lines indicated statistical significance for both men and women, blue lines indicate significance only in men, red lines indicate significance only in women, and orange lines indicated no significance for either men or women. Self-assessed chewing ability was not associated with serum albumin. ADLs were not associated with QOL.

## 4. Discussion

In this study, nutritional status, evaluated by the serum level of albumin, was associated with mortality in women. Self-assessed chewing ability was significantly associated with quality of life (QOL) and the instrumental activity of daily living (IADL) evaluated by the TIMG Index.

The subjects who participated in this study were functionally independent and could attend mass check-ups held at the local city hall or gymnasium. No subjects were hospitalized or living in a nursing home. According to the Kaplan–Meier analysis, their mean life expectancy was 91.28 years for men and 94.38 years for women (Supplementary Table S6). The subjects who participated in this study represented a healthy and long-living population. A previous report showed a large difference in mortality between participants and non-participants in health check-ups [51]. The results of this study may not applicable for hospitalized older adults or older adults residing in nursing homes.

Several studies have suggested that regular diet [52] and nutritional status [53,54,55,56,57] affect the QOL of community-dwelling older adults. Another study showed that chewing ability is significantly greater in subjects with high QOL scores. Dietary intake was not associated with QOL [58]. In this study, chewing ability was significantly associated with two dimensions of QOL. However, nutritional status, as evaluated by the serum level of albumin, and number of remaining teeth were not directly associated with QOL. As shown in Figure 3, the number of remaining teeth is a morphological background factor in oral function [59,60]. Therefore, the number of remaining teeth is not directly associated with QOL. Nutritional status was evaluated by the serum albumin level, which is one of the limitations of this study. A more precise evaluation of nutritional status or regular diet by a validated questionnaire may lead to more precise results. However, these tools were not available when the survey was conducted in 2002.

In this study, the self-assessed chewing ability was associated with three subscales of the TMIG index. The number of remaining teeth and the serum level of albumin were not associated with the IADL. A previous report showed that tooth loss is associated with future decline in higher-level functional capacity [61]. Tooth loss can be compensated for by prosthodontic treatment. In addition, the Japanese national insurance system covers most conventional prosthodontic treatments. Recently, the concept of functional teeth was introduced, and it could be used as a predictor of mortality instead of the number of remaining teeth [62]. One of the limitations of this study is that we did not have data on functional teeth. However, there were only three out of 196 (1.5%) edentulous subjects who did not use dentures.

Recently, the concept of frailty, including oral frailty, has been widely accepted [63,64,65]. Frailty has been evaluated by physical conditions that can be improved by nutritional interventions. For nutritional intervention studies, frail is a more optimal outcome variable than ADLs [66,67,68,69,70]. ADLs do not only describe limited physical conditions. They include other dimensions such as social function and intellectual activity [71]. A previous study showed that physical activity, social role, and mental health are associated with ADLs [41,43,72]. This may be one of the reasons why the serum level of albumin was not significantly associated with the ADL subscales. 

Nutritional factors affect mortality in older adults [73]. In this study, malnutrition was evaluated by the serum level of albumin [74]. A low level of serum albumin is a well-known predictor of mortality in older persons in both the short and long term [74,75,76,77,78,79,80]. The results of this study are consistent with another report conducted in women; however, it was not applicable in men. Except for one subject, all women with less than the cut-off point of albumin died within the observational period. They died within 2000 days. In contrast, one man was alive after the observational period and he became a centenarian. When men and women were combined, the hazard ratio for the serum level of albumin was 1.979 (95% CI: 1.172–3.341, *p* = 0.011).

Self-assessed chewing ability was significantly correlated with QOL and mortality in men. The number of remaining teeth was not statistically significantly correlated with mortality. The subjective method for the evaluation of chewing ability requires a specific device, labor, and costs. Due to its ease of use and cost effectiveness, masticatory dysfunction has generally been assessed by self-assessment-specific questionnaires in epidemiological studies. Studies have shown that the mortality of older adults is influenced by the number of remaining teeth. However, they failed to show a dose–response relationship [81,82]. As mentioned for the QOL, this may be because the effects of tooth loss can be compensated for by the use of proper dentures. In this study, the number of remaining teeth did not directly influence mortality. However, for edentulous subjects, not using dentures was significantly high risk in men (Hazard ratio: 15.160 (*p* = 0.019)). Therefore, tooth loss should be used in combination with the use of dentures [83,84]. Therefore, the concept of functional teeth is reasonable [62]. However, complete denture wearers and subjects with all-natural teeth were treated as equivalent. Further study is necessary to apply the concept of functional teeth in epidemiological studies. The number of remaining teeth should be considered as one of the indicators of oral function. Mortality is a multifactorial issue, and some related factors cause either tooth loss or mortality. In particular, socioeconomic status and health literacy may be important factors in mortality. In this study, we could not obtain these data. It is one of the limitations of this study. However, the association of self-assessed chewing ability with mortality rather than the number of teeth was a reasonable result. Hazard ratio of self-assessed chewing ability for slight hard food was statistically significant for men. It was also significant adjusted by blood tests except for creatinine. Creatinine reflects the muscle activity. Self-assessed chewing ability may reflect the exercise in daily life. However, as shown in Figure 4, clear survival curves were obtained. Self-assessed chewing ability for slight hard food can be the indicator for the prediction of mortality.

There is a sex difference in mortality related to the number of remaining teeth [46,85,86,87,88]. Most studies have shown that tooth loss is a risk factor for mortality in men and not in women [85,87,88]. Other studies have shown contradictory results [46,86]. One report had statistically not significant between men and women [89]. Follow-up periods, the baseline number of remaining teeth, and statistical methods were different between studies. In addition, mortality is a multifactorial issue, and some related factors cause either tooth loss or mortality. Prevalence of noncommunicable diseases may be different between studies. Health care supply system varies from country to country. In addition, the prevalence of noncommunicable diseases may be different between men and women. It is impossible to reach a clear conclusion for the sex difference of mortality. 

Figure 5 shows one of the models. The interactions between health-related factors are different between men and women. When planning a health promotion plan for older adults, different strategies may be necessary for men and women.

## 5. Conclusions

Self-assessed chewing ability was not associated with the serum level of albumin. In dental practice, recovering oral function is not enough for the health promotion of older adults. Additional nutritional instruction is indispensable. Health-related factors were found to interact with each other. However, the interactions were different for men and women. In terms of a health promotion plan for older adults, different strategies are necessary for men and women.

## Figures and Tables

**Table 1:** Correlations of the number of remaining teeth, serum albumin level, and self-assessed chewing ability with quality of life (QOL).

	QOL			
	Function		Role	
	Coefficient (95% CI)	*p*-Value	Coefficient (95% CI)	*p*-Value
Intercept	−0.411 (−1.839–1.018)	0.573	−0.204 (−1.671–1.262)	0.785
Number of remaining teeth	−0.002 (−0.017–0.014)	0.842	−0.006 (−0.022–0.009)	0.437
Serum Albumin (g/dL)	0.106 (−0.243–0.454)	0.553	0.060 (−0.298–0.418)	0.742
Self-assessed Chewing ability	0.336 (0.204–0.469)	<0.001	0.135 (0–0.271)	0.050

The generalized liner model was applied for the factor scores of QOL. Distribution: Normal, Link: Normal. The SF-36 consisted of eight subscales. For these subscales, the generalized linear model was applied. The results are presented in Table S9. Self-assessed chewing ability had a statistically significant correlation with PF, RP, GH, BT, and M, but not with BP, SF, or RE. The number of remaining teeth and serum level of Albumin had no correlations with the eight subscales. CI: confidence interval.

**Table 2:** Correlations of the number of remaining teeth, serum albumin, and self-assessed chewing ability with the IADL.

	TMIG Index					
	Self-Management		Intercultural Activity		Social Role:	
	Coefficient (95% CI)	*p*-Value	Coefficient (95% CI)	*p*-Value	Coefficient (95% CI)	*p*-Value
Intercept	0.153 (−1.234–1.541)	0.828	0.638 (−0.785–2.062)	0.379	0.048 (−1.368–1.464)	0.947
Number of remaining teeth	−0.006 (−0.021–0.009)	0.421	0.013 (−0.002–0.028)	0.100	−0.005 (−0.020–0.010)	0.544
Serum Albumin (g/dL)	−0.015 (−0.354–0.324)	0.930	−0.160 (−0.507–0.188)	0.368	0.006 (−0.340–0.352)	0.974
Self-assessed chewing ability	0.257 (0.129–0.385)	<0.001	0.138 (0.007–0.270)	0.039	0.209 (0.079–0.340)	0.002

The generalized liner model was applied to the factor scores of the TMIG Index. Distribution: Normal, Link: Normal. TMIG index: Tokyo Metropolitan Institute of Gerontology index of competence. IADL: instrumental activity of daily living. CI: confidence interval.

**Table 3:** Hazard ratios of nutritional status, self-assessed chewing ability, and IADL.

		Men		Women	
		Hazard Ratio (95 CI)	*p*-Value	Hazard Ratio (95 CI)	*p*-Value
Nutritional status, Self-assessed chewing ability, number of remaining teeth					
Serum Albumin (<3.7 g/dL/≥3.7 g/dL)		1.294 (0.634–2.641)	0.479	2.621 (1.184–5.803)	0.018
Self-assessed Chewing ability	Moderate hard and hard	1.144 (0.853–1.534)	0.368	1.164 (0.873–1.529)	0.300
	Slight hard	1.821 (1.082–3.049)	0.024	1.149 (0.681–1.946)	0.602
	Easy	1.661 (0.876–3.155)	0.120	0.718 (0.442–1.166)	0.180
Number of remaining teeth		1.028 (0.995–1.060)	0.094	0.966 (0.925–1.009)	0.119
IADL (TMIG Index)					
Self-management (≤4 points/>5 points)		1.212 (0.774–1.898)	0.401	1.548 (0.916–2.616)	0.103
Intellectual activity (≤2 points/>3 points)		2.033 (1.271–3.398)	0.043	1.391 (0.870–2.224)	0.168
Social role (≤2 points/>3 points)		1.569 (0.995–2.475)	0.053	1.345 (0.850–2.130)	0.206

TMIG index: The Tokyo Metropolitan Institute of Gerontology index of competence. CI: confidence interval.

## Supplementary Materials

The following are available online at https://www.mdpi.com/2072-6643/12/11/3315/s1, Figure S1. Diagram of the study design, Figure S2: Item response curve and item information curve of the 15 foods, Table S1: Results of blood tests for the subjects who participated in this study, Table S2: Descriptive statistics of the subscales of the SF 36, Table S3: Factor analysis for the subscales of the SF 36, Table S4: Frequency of items in the TMIG index for subjects who answered yes or able, Table S5: Frequency of scores of the TIMIG Index, Table S6: Factor analysis for the items in the TMIG index, Table S7: Three parameter logistic model of 15 foods, Table S8: Factor analysis for the 15 foods, Table S9: Correlations of number of remaining teeth, serum albumin level, and self-assessed chewing ability with subscales of the QOL, Table S10: Hazard ratios of self-assessed chewing ability of slight hard food adjusted by blood tests, Table S11: Means and medians of survival days.
