# Supplementary material for: Impact of the Serum Level of Albumin and Self-Assessed Chewing Ability on Mortality, QOL, and ADLs for Community-Dwelling Older Adults at the Age of 85: A 15 Year Follow up Study
Source: Nutrients. 2020 Oct 29;12(11):3315. doi: 10.3390/nu12113315 (PMC7692472; doi:10.3390/nu12113315)
Supplement: Supplementary file 1 [file nutrients-12-03315-s001.pdf]

Supplemental materials

Effect of Serum levels albumin and self-assessed chewing ability on QOL, IADL and mortality

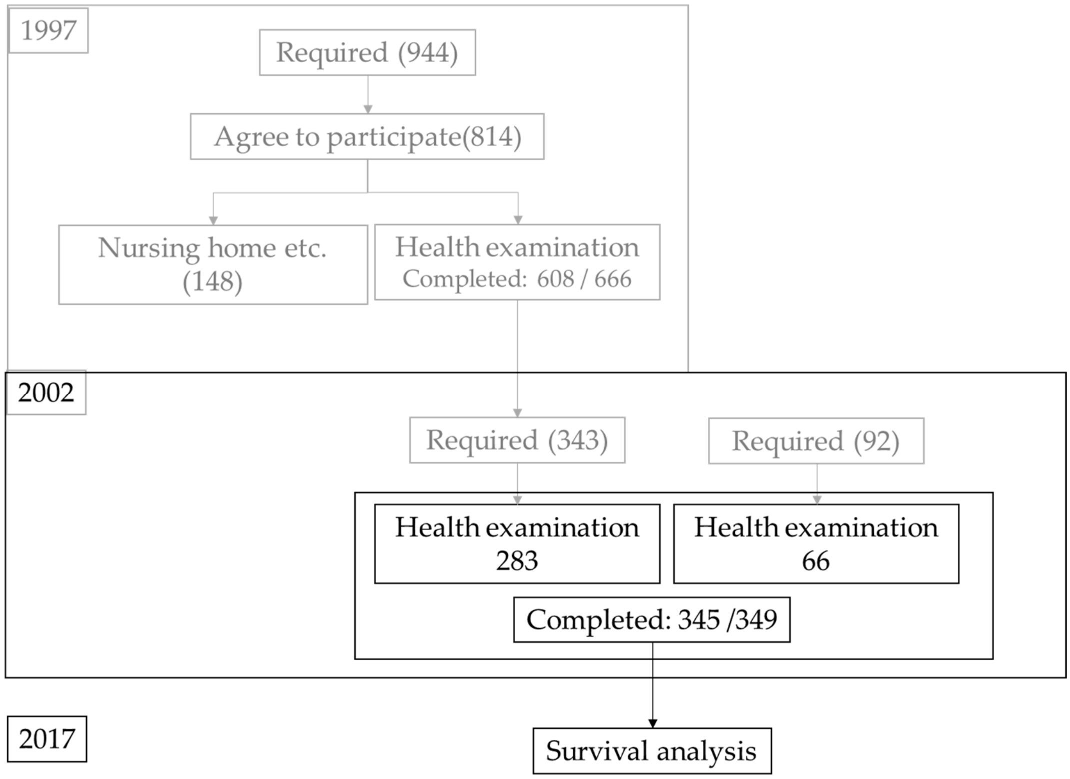

Figure S1. Diagram of the study design

At 2002, 92 subjects lived in two village were additionally rerouted. In this study, data of box in black were used for analysis.

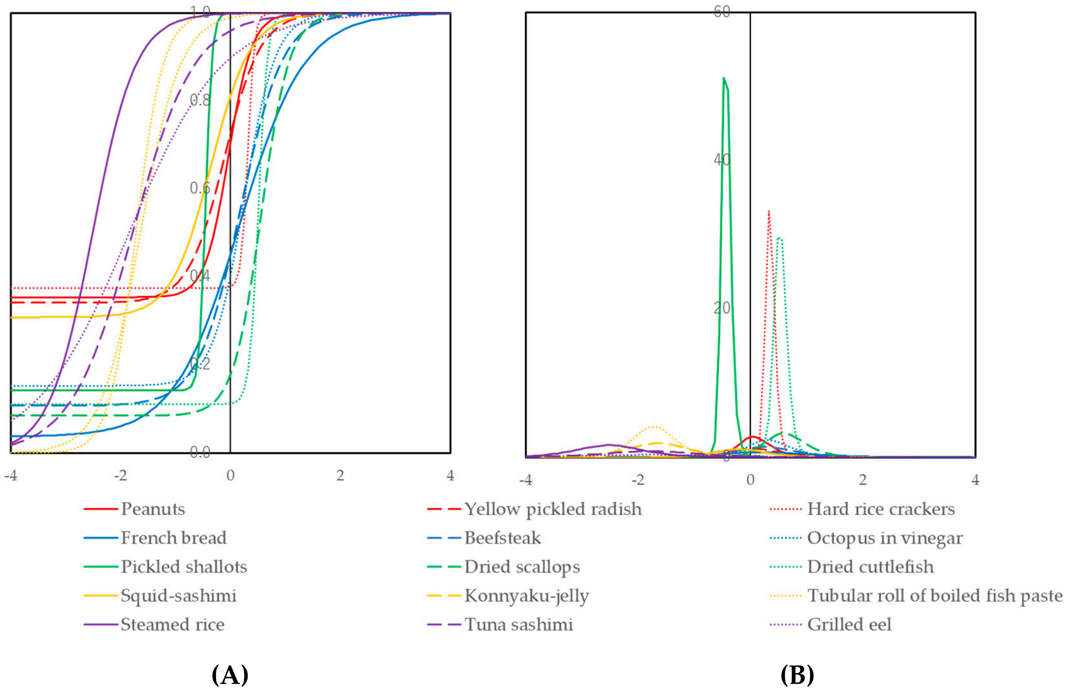

Figure S2. Item response curve and item information curve of the 15 food

Self-assessed chewing ability was evaluated by 15 food. Subjects answered these 15 food chewable or no. Item response curve and item information curve of easy to chew food located backward direction. Those of hard to chew food located forward direction. Dried cuttlefish, hard rice crackers, and pickled shallots had high item information.

Table S1. Results of blood tests of the subjects participated in this study

	Mean +/- SD	Median (25th -75th)
Blood glucose (mg/dL)	128+/-44	115(101-140)
AST (U)	27.9+/-10.5	27(22-31)
ALT (U)	20.9+/-11.6	19(14-23)
γ-GTP (U)	35.1+/-33.6	23(16-39)
Total protein(g/dL)	7.02+/-0.44	7(6.7-7.3)
Albumin	4.01+/-0.31	4(3.85-4.2)
Total cholesterol (mg/dL)	168+/-27	167(151-187)
Try glyceride (mg/dL)	116+/-56	105(74-145)
HDL cholesterol (mg/dL)	49+/-15.5	46(38-55.5)
Creatinine (mg/dL)	0.97+/-0.5	0.9(0.8-1.05)
Ig G (mg/dL)	1463+/-359	1426(1194-1699)
IgA (mg/dL)	336+/-212	286(228-389)
IgM (mg/dL)	77.8+/-36.5	70.5(49.5-97.5)

Abbreviations:

AST Aspartate Aminotransferase

ALT Alanine aminotransferase

γ-GTP γ-glutamyl transpeptidase

HDL High Density Lipoprotein

Table S2. Descriptive statistics of the subscales of SF 36

Subscale	Mean +/- SD	Median (25th -75th)
Physical functioning (PF)	59.3+/-26.2	65(40-80)
Role physical(RP)	66.3+/-41.2	100(25-100)
Bodily pain(BP)	68.7+/-25	72(52-100)
General health(GH)	64.3+/-21.2	65(50-82)
Vitality (VT)	69.7+/-21.7	75(55-90)
Social functioning (SF)	88.3+/-18.7	100(75-100)
Role emotional (RE)	74.1+/-40.1	100(33-100)
Mental health (MH)	78.6+/-17.9	84(68-92)

There values were not normally distributed by Kolmogorov–Smirnov test.

Table S3. Factor analysis for the subscale of SF 36

**Subscale**	**Factor**	
	**1**	**2**
Role physical(RP)	0.976	-0.098
Role emotional (RE)	0.716	-0.082
Bodily pain(BP)	0.525	0.402
Physical functioning (PF)	0.392	0.378
Social functioning (SF)	0.374	0.266
Vitality (VT)	0.436	0.707
Mental health (MH)	0.398	0.600
General health(GH)	0.390	0.592
Sum	2.446	1.689
Percent of variance	30.578	21.118
Cumulative percent	30.578	51.697

Factor analysis was carried out by main principal method with varimax rotation. Subscales of SF 36 were classified into two factors.

Table S4. Frequency of items of TMIG index subjects answered yes or able.

Subscale	Item	n	%
Self-management	Using public transportation	204	59.5
	Shopping	259	75.5
	preparing meals	211	61.5
	Paying bills	250	72.9
	Managing deposit	244	71.1
Intercultural activity	Interest in new story and program about health	205	59.8
	Filling out forms of pension	215	62.7
	Reading newspaper	183	53.4
	Reading books	260	75.8
Social role:	Being called on advice	190	55.4
	Visiting sick friends	174	50.7
	Visiting friends	233	67.9
	Talk to young people	241	70.3

TMIG index: The Tokyo Metropolitan Institute of Gerontology index of competence

Table S5. Frequency of the scores of TIMIG Index

Subscale	Self-management		Intercultural activity		Social role	
**Score**	**n**	%	**n**	%	**n**	%
0	32	9.3	35	10.2	34	9.9
1	27	7.9	53	15.5	46	13.4
2	37	10.8	61	17.8	80	23.3
3	50	14.6	88	25.7	100	29.2
4	68	19.8	106	30.9	83	24.2
5	129	37.6				

Score is a sum of each item, when the subjects answered yes or able.

Score in IADL (≤ 4 points), IA (≤ 2 points), and SR (≤ 2 points) regarded as declined function

TMIG index: The Tokyo Metropolitan Institute of Gerontology index of competence

Table S6. Factor analysis for the items of TMIG index

Subscale	Items	Factor		
		1	2	3
Self-management	Managing deposits	0.668	0.139	0.249
Intellectual activity	Filling out forms of pension	0.660	0.260	0.010
Self-management	Shopping	0.611	0.008	0.304
Self-management	Using public transportation	0.591	0.111	0.327
Self-management	Paying bills	0.526	0.229	0.147
Self-management	Preparing meals	0.471	0.080	0.071
Social Role	Visiting sick friends	0.358	0.269	0.256
Intellectual activity	Reading books	0.058	0.708	0.132
Intellectual activity	Reading newspaper	0.235	0.697	-0.070
Intellectual activity	Interest in new story and program about health	0.104	0.297	0.201
Social Role	Visiting friends	0.116	0.055	0.503
Social Role	Talk to young people	0.129	0.300	0.425
Social Role	Being called on advice	0.272	-0.012	0.359
Sum		2.405	1.399	0.979
Percent of variance		18.503	10.762	7.531
Cumulative percent		18.503	29.266	36.797

Factor analysis was carried out by main principal method with varimax rotation.

TMIG index: The Tokyo Metropolitan Institute of Gerontology index of competence

Table S7. Three parameter logistic model of 15 foods

		Discrimination	Difficulty	Guessing
Very hard-to-chew food	Peanuts	4.69	-0.04	0.35
	Yellow pickled radish	3.04	-0.11	0.34
	Hard rice crackers	16.85	0.31	0.37
Moderately hard-to-chew food	French bread	1.69	0.16	0.04
	Beefsteak	2.61	0.18	0.11
	Octopus in vinegar	3.52	0.24	0.15
	Pickled shallots	17.47	-0.46	0.14
	Dried scallops	3.91	0.55	0.09
	Dried cuttlefish	12.55	0.50	0.11
Slightly hard-to-chew food	Squid-sashimi	2.77	-0.36	0.31
	Konnyaku-jelly	2.76	-1.64	0.00
	Tubular roll of boiled fish paste	4.09	-1.72	0.00
Easy-to-chew food	Steamed rice	2.55	-2.51	0.00
	Tuna sashimi	1.79	-1.79	0.00
	Grilled eel	1.18	-1.85	0.00

Table S8. Factor analysis for the 15 food

Subscale	Food	Factor		
		1	2	3
Very hard-to-chew	Peanuts	0.662	0.202	-0.185
Very hard-to-chew	Yellow pickled radish	0.636	0.235	-0.161
Very hard-to-chew	Hard rice crackers	0.618	0.107	-0.110
Moderately hard-to-chew	French bread	0.517	0.095	0.193
Moderately hard-to-chew	Beefsteak	0.581	0.043	0.352
Moderately hard-to-chew	Octopus in vinegar	0.668	0.080	0.100
Moderately hard-to-chew	Pickled shallots	0.525	0.333	0.192
Moderately hard-to-chew	Dried scallops	0.666	-0.049	0.207
Moderately hard-to-chew	Dried cuttlefish	0.689	-0.036	0.077
Slightly hard-to-chew	Squid-sashimi	0.506	0.157	0.221
Slightly hard-to-chew	Konnyaku-jelly	0.185	0.710	0.089
Slightly hard-to-chew	Tubular roll of boiled fish paste	0.127	0.763	0.183
Easy-to-chew	Steamed rice	0.017	0.656	0.157
Easy-to-chew	Tuna sashimi	0.107	0.445	0.547
Easy-to-chew	Grilled eel	0.060	0.327	0.623
Sum		3.791	2.086	1.132
Percent of variance		25.273	13.906	7.544
Cumulative percent		25.273	39.179	46.723

Factor analysis was carried out by main principal method with varimax rotation.

Table S9. Correlation number of remaining teeth, serum albumin and self-assessed chewing ability with subscales of QOL

Subscale		Coefficient(95% CI)	p-value
Physical functioning (PF)	Intercept	31.517(-5.858-68.893)	0.098
	Number of remaining teeth	0.058(-0.342-0.457)	0.777
	Serum Albumin	6.928(-2.203-16.058)	0.137
	Self-assessed chewing ability	7.754(4.305-11.202)	<0.001
Role physical(RP)	Intercept	53.94(-6.25-114.13)	0.079
	Number of remaining teeth	-0.540(-1.184-0.103)	0.100
	Serum Albumin	3.749(-10.955-18.453)	0.617
	Self-assessed chewing ability	6.485(0.932-12.039)	0.022
Bodily pain(BP)	Intercept	74.823(38.03-111.617)	<0.001
	Number of remaining teeth	0.150(-0.243-0.544)	0.454
	Serum Albumin	-1.648(-10.637-7.342)	0.719
	Self-assessed chewing ability	-0.669(-4.087-2.748)	0.701
General health(GH)	Intercept	61.627(30.176-93.078)	<0.001
	Number of remaining teeth	-0.012(-0.349-0.325)	0.945
	Serum Albumin	0.673(-7.007-8.352)	0.864
	Self-assessed chewing ability	4.613(1.690-7.537)	0.002
Vitality (VT)	Intercept	60.261(29.175-91.347)	<0.001
	Number of remaining teeth	-0.058(-0.391-0.276)	0.734
	Serum Albumin	2.438(-5.153-10.028)	0.529
	Self-assessed chewing ability	5.608(2.744-8.471)	<0.001
Social functioning (SF)	Intercept	91.529(63.913-119.145)	<0.001
	Number of remaining teeth	-0.009(-0.304-0.286)	0.953
	Serum Albumin	-0.772(-7.518-5.975)	0.823
	Self-assessed chewing ability	1.897(-0.651-4.445)	0.144
Role emotional (RE)	Intercept	84.466(24.866-144.065)	0.005
	Number of remaining teeth	-0.337(-0.972-0.299)	0.299
	Serum Albumin	-2.141(-16.695-12.412)	0.773
	Self-assessed chewing ability	2.007(-3.466-7.480)	0.472
Mental health (MH)	Intercept	76.48(50.654-102.305)	<0.001
	Number of remaining teeth	0.158(-0.119-0.435)	0.264

	Serum Albumin	0.378(-5.928-6.684)	0.907
	Self-assessed chewing ability	5.911(3.533-8.290)	<0.001

SF-36 consisted of 8 subscales. For these subscales, generalized linear model was applied. Distribution: Normal, Link: Normal. Self-assessed chewing ability had statistically significant correlation with PF, RP, GH, BT, and MH, but not BP, SF, RE. Number of remaining teeth and serum levels Albumin had no correlation with the 8 subscales.

Table S10. Hazard ratios of self-assessed chewing ability of slight hard food adjusted by blood tests

	Men		Women	
	Hazard ratio(95% CI)	P-value	Hazard ratio(95% CI)	P-value
Blood glucose (mg/dL)	0.997(0.992-1.002)	0.286	1.006(1.000-1.012)	0.048
Self-assessed Chewing ability (Slight hard)	1.821(1.073-3.090)	0.026	1.235(0.662-2.307)	0.507
AST (U)	1.006(0.989-1.023)	0.507	1.008(0.983-1.033)	0.538
Self-assessed Chewing ability (Slight hard)	1.924(1.136-3.257)	0.015	1.340(0.760-2.361)	0.312
γ-GTP (U)	1.003(0.997-1.009)	0.279	1.014(0.998-1.030)	0.089
Self-assessed Chewing ability (Slight hard)	1.929(1.137-3.272)	0.015	1.412(0.796-2.502)	0.238
Total protein(g/dL)	0.645(0.359-1.158)	0.142	1.384(0.787-2.434)	0.259
Self-assessed Chewing ability (Slight hard)	1.836(1.08-3.121)	0.025	1.38(0.784-2.430)	0.264
Total cholesterol (mg/dL)	0.997(0.986-1.007)	0.526	0.997(0.988-1.006)	0.481
Self-assessed Chewing ability (Slight hard)	1.911(1.126-3.243)	0.016	1.347(0.764-2.373)	0.303
Try glyceride (mg/dL)	0.999(0.995-1.003)	0.500	0.998(0.994-1.002)	0.331
Self-assessed Chewing ability (Slight hard)	0.994(0.975-1.013)	0.545	1.003(0.986-.1.021)	0.742
HDL cholesterol (mg/dL)	1.347(0.765-2.470)	0.302	1.919(1.131-3.255)	0.016
Self-assessed Chewing ability Slight hard	1.869(1.102-3.17)	0.020	1.308(0.739-2.316)	0.357
Creatinine (mg/dL)	2.637(0.900-7.723)	0.077	5.986(2.414-14.843)	<0.001
Self-assessed Chewing ability (Slight hard)	1.574(0.869-2.851)	0.134	1.422(0.804-2.517)	0.227
Ig G (mg/dL)	1.000(0.999-1.000)	0.464	1.001(1.000-1.001)	0.103
Self-assessed Chewing ability (Slight hard)	1.917(1.133-3.244)	0.015	1.297(0.691-2.432)	0.418
IgA (mg/dL)	1.001(0.999-1.002)	0.478	1.001(0.999-1.003)	0.344
Self-assessed Chewing ability (Slight hard)	1.882(1.111-3.189)	0.019	1.259(0.672-2.360)	0.473
IgM (mg/dL)	0.998(0.991-1.004)	0.499	1.001(0.995-1.007)	0.745
Self-assessed Chewing ability (Slight hard)	1.876(1.106-3.182)	0.020	1.218(0.651-2.277)	0.537

Table S11. Mean and median of survival days

				Estimation		P-value		
				Mean(95%CI)	Median (95%CI)	Log Rank (Mantel-Cox)	Breslow (Generalized Wilcoxon)	Tarone-Ware
Men				2291(1988-2595)	1959(1800-2118)			
Women				3424′(3071-3778)	3431(2413-4449)			
Serum Albumin		Men	<3.7g/dL	1737(870-2605)	1253(734-1772)	0.475	0.071	0.110
			>= 3.7g/dL	2422(2090-2753)	2033(1770-2296)			
		Women	<3.7g/dL	3003(1648-4359)	1822(0-3660)	0.014	0.004	0.005
			>= 3.7g/dL	3630(3255-4004)	3771(2993-4549)			
Chewing ability	Konnyaku-jelly	Men	Not chewable	1312(968-1656)	1153(812-1494)	0.060	0.091	0.074
			Chewable	2355(2035-2674)	2024(1869-2179)			
		Women	Not chewable	2686(1670-3703)	2918(405-5431)	0.414	0.361	0.374
			Chewable	3461(3095-3828)	3747(2766-4728)			
	Tubular roll of boiled fish paste	Men	Not chewable	1284(731-1838)	1007(991-1023)	0.046	0.101	0.069
			Chewable	2330(2017-2643)	2024(1869-2179)			
		Women	Not chewable	3112(2054-4169)	2918(451-5385)	0.868	0.803	0.826
			Chewable	3424(3058-3789)	3431(2329-4533)			
	Steamed rice	Men	Not chewable	1063(953-1173)	1007	0.034	0.073	0.050
			Chewable	2324(2013-2634)	2005(1836-2174)			
		Women	Not chewable	2343(1041-3645)	2918	0.345	0.635	0.506
			Chewable	3454(3093-3814)	3747(2758-4736)			
Teeth		Men	Edentulous	1950(1552-2349)	1488(873-2103)	0.013	0.008	0.009
			Dentate	2658(2212-3105)	2163(1703-2623)			
		Women	Edentulous	3603(3199-4007)	3934(3076-4792)	0.431	0.162	0.205
			Dentate	3063(2328-3797)	2245(1742-2748)			
TIMG Index	Self-management	Men	<= 4 point	2425(2001-2848)	2163(1617-2709)	0.398	0.369	0.323
			> 5point	2130(1694-2566)	1849(1526-2172)			
		Women	<= 4 point	3907(3286-4528)	4475(2625-6325)	0.100	0.055	0.060
			> 5point	3210(2788-3632)	3076(2383-3769)			
	Intellectual activity	Men	<= 2 point	2512(2155-2869)	2033(1585-2481)	0.006	0.006	0.004
			> 3 point	1593(1132-2054)	1168(771-1565)			
		Women	<= 2 point	3682(3168-4195)	4070(3144-4996)	0.166	0.211	0.183
			> 3 point	3210(2731-3690)	2918(961-4875)			
	Social role	Men	<= 2 point	2526(2138-2914)	2163(1736-2590)	0.050	0.011	0.014
			> 3 point	1950(1460-2440)	1450(1035-1865)			
		Women	<= 2 point	3636(3172-4100)	3771(2374-5168)	0.204	0.028	0.060
			> 3 point	3193(2658-3727)	3419(2054-4784)			

Life expectancy of men can be calculated by transformation of days to years. Mean life expectancy for men was 91.28 years old for men and 94.38 years old for women.
